# Origin of Chinese Goldfish and Sequential Loss of Genetic Diversity Accompanies New Breeds

**DOI:** 10.1371/journal.pone.0059571

**Published:** 2013-03-19

**Authors:** Shu-Yan Wang, Jing Luo, Robert W. Murphy, Shi-Fang Wu, Chun-Ling Zhu, Yun Gao, Ya-Ping Zhang

**Affiliations:** 1 State Key Laboratory of Genetic Resources and Evolution, and Yunnan Laboratory of Molecular Biology of Domestic Animals, Kunming Institute of Zoology, the Chinese Academy of Sciences, Kunming, China; 2 School of Life Sciences, University of Science and Technology of China, Hefei, China; 3 Laboratory for Conservation and Utilization of Bio-resources, College of Life Sciences, Yunnan University, Kunming, China; 4 Centre for Biodiversity and Conservation Biology, Royal Ontario Museum, Toronto, Canada; BiK-F Biodiversity and Climate Research Center, Germany

## Abstract

**Background:**

Goldfish, *Carassius auratus*, have experienced strong anthropogenic selection during their evolutionary history, generating a tremendous extent of morphological variation relative to that in native *Carassius*. To locate the geographic origin of goldfish, we analyzed nucleotide sequences from part of the control region (CR) and the entire cytochrome *b* (*Cytb*) mitochondrial DNA genes for 234 goldfish and a large series of native specimens. Four important morphological characteristics used in goldfish taxonomy–body shape, dorsal fin, eye shape, and tailfin–were selected for hypothesis-testing to identify those that better correspond to evolutionary history.

**Principal Finding:**

Haplotypes of goldfish rooted in two sublineages (C5 and C6), which contained the haplotypes of native *C. a. auratus* from southern China. Values of *F*_ST_ and N_m_ revealed a close relationship between goldfish and native *C. a. auratus* from the lower Yangtze River. An extraordinary, stepwise loss of genetic diversity was detected from native fish to goldfish and from Grass-goldfish relative to other breeds. Significantly negative results for the tests of Tajima’s *D* and Fu and Li’s D* and F* were identified in goldfish, including the Grass breed. The results identified eye-shape as being the least informative character for grouping goldfish with respect to their evolutionary history. Fisher’s exact test identified matrilineal constraints on domestication.

**Conclusions:**

Chinese goldfish have a matrilineal origin from native southern Chinese *C. a. auratus*, especially the lineages from the lower Yangtze River. Anthropogenic selection of the native *Carassius* eliminated aesthetically unappealing goldfish and this action appeared to be responsible for the stepwise decrease in genetic diversity of domesticated goldfish, a process similar to that reported for the domestication of pigs, rice, and maize. The three-breed taxonomy–Grass-goldfish, Egg-goldfish, and Wen-goldfish–better reflected the history of domestication.

## Introduction

Goldfish, one of the first animals domesticated for ornamental purposes, has experienced extreme anthropogenic selection during its evolutionary history to create aesthetically appealing forms [1], [2]. Widely distributed across Eurasia [3], native feral *Carassius* (crucian carp) can naturally change their body color from gray to red [1], [2]. Feral red goldfish are thought to be the ancestral forms of Chinese goldfish [1], [2].

The ability to change color has led to aquiculture of the fish for use in religion [4]. The earliest record of anthropogenic usage dates to the Tsin Dynasty (265–419 A.D.) of China as noted in the Compendium of Materia Medica [4]. Strong anthropogenic selection during cultivation is likely responsible for much of the phenotypic variation seen today [1], [2], [4]. Some goldfish possess features such as egg-shaped bodies, celestial or telescopic eyes, fancy tailfins, lionhead morphotypes, a raspberry-like hood encasing the head (oranda), no dorsal fin, and other variants [2], [5]. Around 1502 A.D. goldfish were exported to Japan [1] and around 1700 A.D. to Europe [1], [2], [5].

The tremendous extent of morphological variation in goldfish owing to anthropogenic selection causes difficulties in evolutionary taxonomy. Linnaeus (1758) originally named the goldfish as *Cyprinus auratus* because morphologically it is similar to the common carp, *Cyprinus carpio* [6]. Subsequently, *Cyprinus auratus* was transferred to the genus *Carassius* as *Carassius auratus* [6]. Several taxonomic schemes (Table 1) exist in China for goldfish, each of which focuses on different morphological features [4], [7]–[9]. Most frequently, three terms are used to designate breeds: Grass-goldfish, Wen-goldfish, and Egg-goldfish. Assignment of an individual fish to one breed or another depends on body-shape (slender or egg-shaped) and condition of the dorsal fin (retained or loss) [7]. Systems involving four (Grass-goldfish, Wen-goldfish, Egg-goldfish, and Dragon-eye-goldfish) [8] and five (Grass-goldfish, Wen-goldfish, Egg-goldfish, Dragon-eye-goldfish, and Dragon-dorsal-goldfish) breeds are based on eye-shape (dragon or normal) as well as the two previous morphological characteristics [9]. Extended celestial or telescope eyes distinguish the Dragon-eye-goldfish from those with normal eyes [8], [9]. Thus, the Dragon-dorsal-goldfish has dragon-eyes and no dorsal fin [9]. Although the numbers of tailfins (single or double) is not a standard for grouping goldfish, it is an important morphological characteristic used to describe the breeds [4], [7]–[9]. The detailed descriptions of the previous four morphological characteristics for the three taxonomic schemes are listed in Table 1. Taxonomy is more informative when based on evolutionary history, but these various taxonomies (Table 1) focus only on human-selected morphological characteristics and likely obscure history. Unfortunately, no written history details the sequential development of the breeds. This necessitates a reassessment of goldfish-taxonomy to assure it better mirrors evolutionary history, and not merely the extent of morphological divergence.

**Table 1:** Features of different breed-systems of goldfish based on morphology.

Morphology	Three-breed system	Four-breed system	Five-breed system
Body-shape (slender vs. egg-shaped)	Grass- (slender); Wen- (Egg-shaped); Egg- (Egg-shaped)	Grass- (slender); Wen- (Egg-shaped); Egg- (Egg-shaped); Dragon- (Egg-shaped)	Grass- (slender); Wen- (Egg-shaped); Egg- (Egg-shaped); Dragon- (Egg-shaped); Dragon-dorsal- (Egg-shaped)
Condition of dorsal fin (retained vs. loss)	Grass- (retained); Wen- (retained); Egg- (loss)	Grass- (retained); Wen- (retained); Egg- (loss); Dragon- (retained or loss)	Grass- (retained); Wen- (retained); Egg- (loss); Dragon- (retained); Dragon-dorsal- (loss)
Eye shape (normal vs. extended)	Grass- (normal); Wen- (normal or extended); Egg- (normal or extended)	Grass- (normal); Wen- (normal or extended); Egg- (normal or extended); Dragon- (extended)	Grass- (normal); Wen- (normal or extended); Egg- (normal); Dragon- (extended)Dragon-dorsal- (extended)
Numbers of tailfins(single vs. double)	Grass- (single); Wen- (single or double); Egg- (double)	Grass- (single); Wen- (single or double); Egg- (double); Dragon- (single or double)	Grass- (single); Wen- (single or double); Egg- (double); Dragon- (single or double); Dragon-dorsal- (double)

Chen [10] reported reproductive viability in hybrids between breeds of goldfish and native *Carassius*. The muscle proteins of native *Carassius* are similar to those of goldfish [11]. Analyses of nucleotide sequence data from partial fragments of mitochondrial DNA (mtDNA) control region (CR; 471 bp), also known as the D-loop, obtain the same conclusion [12]. Komiyama *et al*. [13] analyzed a portion of the mitochondrial genome (740 bp) from 67 specimens of *Carassius* including 44 specimens of goldfish, and further 11180 bp from seven specimens of goldfish. Although they evaluated most of the mitochondrial genome, their sampling on mainland China was limited. Their matrilineal history hypothesizes that the ancestral breed is the Gibelio group of Chinese *Carassius*. Our prior research on the biogeography of the East Asian *C. auratus* complex used 1876 partial CR (426 bp) and 187 complete cytochrome *b* (*Cytb*; 1140 bp) gene sequences from 67 localities representing most of the species’ range and identified three distinct, mostly geographically constrained matrilines [14]. These analyses provide an opportunity to investigate the origin and domesticated history of goldfish from the perspective of the large population of native *Carassius*.

Herein, we reconstruct the matrilineal relationships using de novo sequences of goldfish as well as mtDNA data of wild *Carassius* from prior studies [14]. Analyses are used to infer the geographic origin of goldfish and to investigate the genetic consequences of extreme anthropogenic selection. We use CR because of its high mutation rate, which facilitates the resolution of intraspecific matrilineal relationships [15]–[18]. We also employ nucleotide sequences of *Cytb* because this gene is less subject to substitution-saturation, which makes it more reliable than CR for evaluating interspecific relationships [15], [17]–[20]. Furthermore, we select several important morphological characteristics of goldfish taxonomy in China for hypothesis-testing to identify those that better correspond to evolutionary history.

## Materials and Methods

### Ethics Statement

All samples of fish from China used in this study were obtained and handled following the guidelines of the by-laws on experimentation on animals, and was approved by the Ethics and Experimental Animal Committee of Kunming Institute of Zoology, Chinese Academy of Science, China (KIZ_YP201002).

### Sampling and Molecular Methods

One hundred and ninety specimens of goldfish were collected from Hangzhou (37 specimens), Kunming (56 specimens), Changchun (20 specimens), Lanzhou (23 specimens) and Beijing (22 specimens), China, Seoul (11 specimens), South Korea, and Toronto (21 specimens), Canada. In addition, 5 sequences for Chinese goldfish [13], 39 sequences for Japanese goldfish [13] as well as 1876 sequences for native *Carassius* with detailed sampling localities and haplotypes [14] were obtained from GenBank (Table 2). Samples used for morphological analyses were photographed and then stored as voucher specimens. Either tailfin clips or muscle tissue samples were collected from individuals and stored at −20°C until processing. Genomic DNA from freshly frozen or ethanol-fixed tissues was extracted by the standard phenol/chloroform method.

**Table 2:** Summary statistics for the CR and *Cytb* markers used in the study.

Lineages	N	V	P	N_H_	H_d_	π	*D*	*D* *	*F* *
**CR (426 bp)**									
*Carassius carassius*	9	2	0	3	0.4167±0.1907	0.0030±0.0024	−1.362	−1.505	−1.626
Native *Carassius auratus* complex	1867	98	86	208	0.9673±0.0017	0.1564±0.0746	0.169	−1.218	−0.628
Lineage A: *C. a. cuvieri*	5	1	0	3	0.7000±0.2184	0.0019±0.0018	−0.817	−0.816	−0.772
Lineage B: *C. a. langsdorfii*	421	42	37	46	0.9531±0.0032	0.0560±0.0272	0.665	0.471	0.674
Sublineage C1	62	16	16	15	0.8636±0.0266	0.0129±0.0070	−0.153	0.679	0.461
Sublineage C2: *C. a. gibelio*	243	26	19	30	0.5425±0.0387	0.0152±0.0080	−1.678	−2.008	−2.262
Sublineage C3	60	16	6	9	0.7994±0.0299	0.0132±0.0071	−1.308	−2.999	−2.860
Sublineage C4	277	17	16	18	0.5012±0.0358	0.0513±0.0251	−0.475	0.073	−0.163
Sublineage C5 and C6: *C. a. auratus*	799	57	47	87	0.9397±0.0040	0.0929±0.0447	−1.336	−0.879	−1.323
Goldfish	234	11	8	9	0.3782±0.0401	0.0023±0.0018	−1.083	−0.871	−1.139
Grass-goldfish	82	10	7	6	0.4436±0.0635	0.0045±0.0029	−0.162	−0.636	−0.560
Egg-goldfish	60	2	2	4	0.6471±0.0458	<0.0001	0.964	0.734	0.933
Wen-goldfish	92	2	1	3	0.1949±0.0667	<0.0001	−0.993	−1.059	−1.214
***Cytb*** ** (1140 bp)**									
*Carassius carassius*	4	0	0	1	\	\	\	\	\
Native *Carassius auratus* complex	187	224	184	103	0.9806±0.0044	0.0257±0.0125	−0.765	0.314	−0.634
Lineage A: *C. a. cuvieri*	2	1	0	2	\	\	\	\	\
Lineage B: *C. a. langsdorfii*	24	80	44	20	0.9855±0.0159	0.0154±0.0079	−0.704	−1.109	−1.152
Sublineage C1	15	37	27	12	0.9714±0.0327	0.0092±0.0050	−0.345	−2.060	−2.180
Sublineage C2: *C. a. gibelio*	19	14	8	10	0.9064±0.0446	0.0030±0.0018	−0.493	−0.628	−0.683
Sublineage C3	9	10	5	4	0.6944±0.1470	0.0028±0.0018	−0.649	−0.306	−0.434
Sublineage C4	32	29	10	10	0.7258±0.0749	0.0050±0.0027	−0.757	−**2.700** *	−2.434
Sublineage C5 and C6: *C. a. auratus*	84	64	40	45	0.9541±0.0149	0.0062±0.0033	−1.573	−**2.736** *	−**2.726** *
Goldfish	180	25	6	8	0.1971±0.0384	0.0007±0.0006	−**2.266** **	−**6.423** **	−**5.703** **
Grass-goldfish	65	25	6	7	0.4509±0.0684	0.0017±0.0011	−**1.943** *	−**4.737** **	−**4.423** **
Egg-goldfish	34	0	0	1	\	\	\	\	\
Wen-goldfish	81	1	0	2	0.0230±0.0022	<0.0001	−1.043	−1.995	−1.992

N, number of sequences; V, variable sites of sequences; P, potentially parsimony-informative sites of sequences; N_H_, number of haplotypes; H_d_, haplotype diversity; π, nucleotide diversity; *D*, Tajima’s D-statistic; D*, Fu and Li’s D- statistic; F*, Fu and Li’s F- statistic;

* *P*<0.05.

** *P*<0.01.

PCR amplifications were performed in a total volume of 50 µl containing 1x buffer, 0.15 mM MgCl_2_ (Sina-American, Beijing, China), 0.25 mM dNTPs (Amersco, Solon, OH, USA), 1 U Taq DNA polymerase (Sina-American) and 25–50 ng total DNA. Primers for amplification were identical to those of Gao *et al*. [14]. Amplifications were performed on a Gene Amp PCR system 9700 (Applied Biosystems, Foster City, CA, USA) following the conditions: pre-denaturation at 96°C for 2 min followed by 30 cycles of denaturation-annealing-elongation (96°C, 1 min; CR: 58°C and *Cytb*: 50°C, 1 min; 72°C, 1 min) and a final extension at 72°C for 10 min. The corresponding PCR products were purified on agarose gels and extracted (Watson BioMedical Inc., Shanghai). Sequencing reactions were run on a 3730 (ABI) with ABI PRISM BigDye Terminator Cycle Sequencing Ready Reaction Kit (ABI) following the manufacturer’s recommendations. All PCR products were sequenced in both directions.

### Analyses of Sequence Data

Sequences were assembled using DNASTAR v.5.0 (DNASTAR Inc., Madison, WI, USA) and manually verified. Sequence alignments and information on nucleotide variation were obtained using MEGA v.4.0 [21], [22]. DAMBE v.4.1.19 [23] was used to identify shared haplotypes. The new haplotypes identified from goldfish, plus the 180 haplotypes of combined CR and *Cytb* from a prior study [14], were used for the new reconstruction of the matrilineal genealogy.

Phylogenetic analyses were conducted using maximum likelihood (ML) and maximum parsimony (MP) in PAUP* 4.0b10 [24], and Bayesian inference (BI) in MrBayes v.3.0b4 [25]. All analyses were based on the concatenated *Cytb* and CR data. Likelihood ratios tests [26]–[28] implemented in MODELTEST v.3.7 [29] were employed to select the best-fitting models for the ML and BI analyses. The GTR+I+G model was selected for the combined dataset by the Akaike Information Criterion [30]. In the ML and MP analyses, a heuristic search with 100 random additions replicates was involved. BI used four simultaneous Metropolis-coupled Monte Carlo Markov chains running for 5,000,000 generations. Convergence to stationarity was evaluated by TRACER v.1.5 [31] using log-likelihood values. The first 50% of the trees were discarded as burn-in and the remaining tree samples were used to generate a consensus tree. Nodal support for the ML and MP tree building methods was assessed using nonparametric bootstrapping (BS) [32] calculated in PAUP* for the MP analysis (MPBS) and RAxML [33] for ML (MLBS) using 1000 pseudoreplicates each. Bayesian posterior probability (BPP) values, the frequency of nodal resolution in the majority rule consensus tree, were calculated through the BI analysis, and the BS for each node in MP as well as ML reconstructions were plotted on the tree.

According to the historical distribution of East Asian *Carassius* [3], [34]–[38], we classified the 67 sampling localities for native *Carassius* from a prior study [14] into four geographic regions: northern China (NC), southern China (SC), Japan (JA), and Europe (Russia and Czech Republic; EU). Sampling localities for native *Carassius* in China [14] were further classified into eight regions according to the distribution of Chinese drainages: Yunnan-Guizhou Plateau, Yellow River, Pearl River, middle Yangtze River, lower Yangtze River, Minjiang River, Amur River, and inland rivers in Xinjiang. We used ARLEQUIN v.3.1 [39] to calculate pairwise *F*_ST_ values and the number of migrants in each generation (N_m_) based on the CR datasets. The analyses identified the extent of divergence and inferred gene flow between the native *Carassius* from these regions and goldfish. An analysis of molecular variance (AMOVA), implemented in ARLEQUIN, was used to evaluate the genetic divergence in goldfish from southern China (Hangzhou and Kunming), northern China (Changchun, Lanzhou and Beijing), Canada, South Korea and Japan.

Haplotype diversity (H_d_) and nucleotide diversity (π) values for the different lineages of native *Carassius* and breeds of Chinese goldfish were calculated based on CR and *Cytb* datasets respectively using ARLEQUIN.

The possible effects of demographic events during goldfish domestication were examined by the tests of Tajima’s *D* [40] and Fu and Li’s [41] D* and F* based on CR and *Cytb* separately. These tests were performed using DNASP v.5.00 [42].

### Hypothesis-tests of Morphology and Genealogy

We tested for the correspondence between the three methods of morphologically grouping Chinese goldfish and genealogical history based on the concatenated dataset. The morphological data consisted of the four characters traditionally used for Chinese goldfish taxonomy (Table 1): body-shape (slender or egg-shaped), presence or absence of the dorsal fin, the eye shape (normal or derived), and single versus double tailfins. We identified specific haplotypes that were constrained for one morphological condition, and *C. carassius* was used as the outgroup taxon to determine character state polarities. For example, we evaluated whether the specific haplotypes for slender body-shape was constrained to one matriline, or not. The morphology-based trees were compared to the best unconstrained molecular tree. A MP molecular tree that represented a particular morphological topology was estimated using constrained tree searches in PAUP*, and a heuristic search with 100 random additions replicates was involved for each analysis. Each of the constrained trees was compared to the unconstrained MP topology using a non-parametric Templeton test [43] in PAUP*. Constrained and unconstrained topologies were similarly calculated under the ML criterion in a heuristic search with 100 random additions replicates and compared using the Shimodaira and Hasegawa [44] test (SH) implemented in PAUP*.

A Fisher’s exact test was used to examine the matrilineal distribution of goldfish based on the concatenated *Cytb* and CR datasets. DNASP [42] calculated the polymorphic sites (variable and potentially parsimony-informative sites) for native *Carassius* and goldfish, respectively, and the Fisher’s exact test was performed using SPSS version 13.0.

## Results

### Haplotype Nomenclature

To avoid confusion, we employed a nomenclature to distinguish haplotypes obtained from the two genes. Haplotypes starting with ‘h’ were used to denote CR data, those with the prefix “Jap” were CR sequences unique to Japanese goldfish, and those starting with “B” indicated *Cytb* data only. The designations were combined to indicate total mtDNA variation. Accordingly, a haplotype consisting of CR haplotype h13 and *Cytb* haplotype B10 was termed h13B10.

### Sequence Variation

Sequence variation in CR and *Cytb* markers of goldfish was summarized in Table 2. The CR sequences (426 bp) of goldfish contained only 11 variable sites of which eight were potentially parsimony-informative. Analyses identified nine haplotypes from 234 specimens. Two haplotypes (Jap1, Jap2) were unique to goldfish, and seven were shared with native *Carassius*. Haplotype h20 was most common in goldfish, being shared by 181 specimens. Among the 1140 bp of *Cytb* data, 25 sites exhibited variation and among these only six sites were potentially parsimony-informative. For *Cytb*, eight haplotypes were identified from the 180 sequences of goldfish, of which three (B105–B107) were not shared with native *Carassius*. Shared by 160 specimens, haplotype B13 was the most common one. Combined, the CR and *Cytb* data identified 12 haplotypes. Seven of these haplotypes–h1_2B13, h20B13, h55B105, h55B106, h56B107, Jap1B13, and Jap2B13–were unique to goldfish. One hundred and forty seven specimens of goldfish shared the most common haplotype, h20B13; this haplotype was not shared with native *Carassius*. Only five haplotypes–h1_2B22, h13B10, h19B13, h32B25, and h56B9–were shared with native *Carassius*. More specimens of native *Carassius* were sequenced for CR (1876) than *Cytb* (187) [14] and this may have resulted in the resolution of a greater number of haplotypes unique to goldfish for *Cytb*.

### Matrilineal History

**Figure 1:**
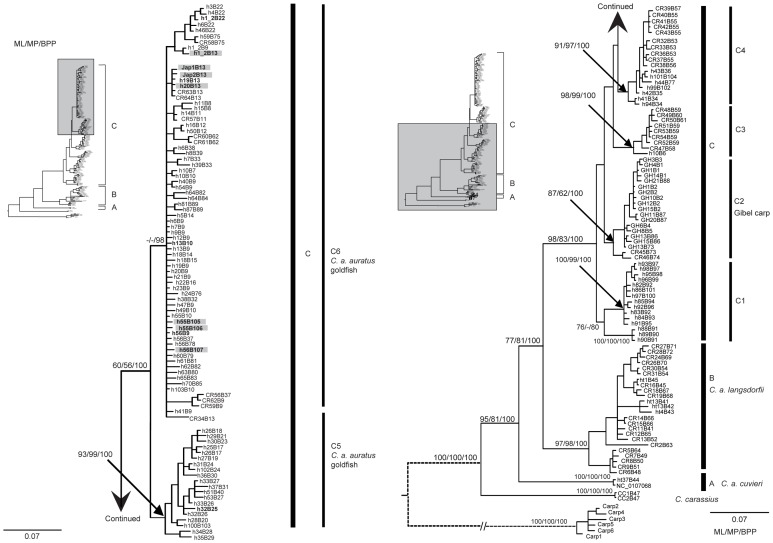
Matrilineal genealogy. The genealogical tree based on Bayesian inference for 180 combined mtDNA cytochrome *b* and partial control region sequences from Gao *et al*. [14] plus seven new unique haplotypes identified herein from goldfish. Numbers above the branches represent bootstrap branch support (>50%) for maximum likelihood and maximum parsimony estimations and Bayesian posterior probabilities, respectively. Haplotypes for goldfish were represented with bold font. Haplotypes in a gray square are unique for goldfish.

Bayesian inference (BI), maximum likelihood (ML), and maximum parsimony (MP) analyses of the concatenated CR and *Cytb* sequences yielded the same topology (Fig. 1) and this was the same as that of Gao *et al*. [14]. Haplotypes identified in *C. a. cuvieri* (Lineage A) and *C. a. langsdorfii* (Lineage B) were not found in goldfish. All haplotypes identified in the gibel carp (a.k.a. Gibelio) clustered together forming sublineage C2; none of these haplotypes was detected in goldfish. Haplotypes identified from goldfish occurred in sublineages C5 and C6 (Fig. 1) only. In our genealogy, sublineages C5 (MLBS = 93%, MPBS = 99%, BPP = 100%) and C6 (BPP = 98%) clustered together with high support (MLBS = 60%, MPBS = 56%, BPP = 100%), both containing the haplotypes identified in native *C. a. auratus* that generally occurred in southern China [14]. Sublineage C5 contained native *C. a. auratus*, fish especially associated with the Yangtze River [14]. Eleven of 12 haplotypes identified from goldfish clustered in sublineage C6, and only one haplotype (h32B13) from Grass-goldfish located in sublineage C5.

### Genetic Divergence between Native *Carassius* and Goldfish

The *F*_ST_ value between goldfish and native *Carassius* from southern China (SC) was substantially lower (0.2157) than that between goldfish and native *Carassius* from northern China (NC; 0.9958), Europe (EU; 0.8942), or Japan (JA; 0.7461). Values of N_m_ also indicated more gene flow between the goldfish and native *Carassius* from SC (2.9847) than between goldfish and the native *Carassius* from NC (0.0254), EU (0.1337), and JA (0.2922). Values of *F*_ST_ and N_m_ between the native *Carassius* from eight Chinese drainages and goldfish were also calculated (Table 3). Native *Carassius* from the lower Yangtze River and goldfish had the lowest *F*_ST_ value (0.2829). Values of N_m_ indicated strong gene flow between goldfish and native *Carassius* from the lower Yangtze River (N_m_ = 1.2674) and Yunnan-Guizhou Plateau (N_m_ = 1.2580).

**Table 3:** Pairwise *F*_ST_ values and the numbers of migration in every generation (N_m_) between the wild *Carassius* from eight Chinese drainages and goldfish; pairwise *F*_ST_ values were below the diagonal; the numbers of migration in every generation (N_m_) were above the diagonal.

	Lower Yangtze River	Yunnan-GuizhouPlateau	Yellow River	Pearl River	Middle YangtzeRiver	Minjiang River	Inland rivers in Xinjiang	Amur River	Goldfish
Lower Yangtze River		4.3225	7.1872	7.8107	1.3567	0.3763	0.4643	0.2960	1.2674
Yunnan-Guizhou Plateau	0.1037		9.5389	23.6806	1.1782	0.3062	0.4832	0.3305	1.2580
Yellow River	0.0650	0.0498		26.2527	1.7216	0.3130	0.5190	0.3063	0.6835
Pearl River	0.0602	0.0207	0.0187		2.5077	0.3860	0.7322	0.4001	0.6376
Middle Yangtze River	0.2693	0.2979	0.2251	0.1662		0.5883	3.4655	1.4651	0.3130
Minjiang River	0.5706	0.6202	0.6154	0.5643	0.4594		0.3881	0.3014	0.1027
Inland rivers in Xinjiang	0.5185	0.5086	0.4907	0.4058	0.1261	0.5630		17.8523	0.1025
Amur River	0.6282	0.6020	0.6201	0.5555	0.2544	0.6234	0.0272		0.0908
Goldfish	0.2829	0.2844	0.4225	0.4395	0.6125	0.8296	0.8299	0.8463	

We classified the goldfish by the three-breed taxonomy and subsequently compared the divergence between these breeds and native *C. a. auratus*. For the CR dataset, the *F*_ST_ value between southern Chinese *C. a. auratus* (sublineages C5 and C6) and Grass-goldfish (0.0591) was lower than that between either Egg-goldfish (0.1355) or Wen-goldfish and southern Chinese *C. a. auratus* (0.1164). The N_m_ value between southern Chinese *C. a. auratus* and Grass-goldfish (6.5712) was higher than between Egg-goldfish (3.0126) or Wen-goldfish (3.4842) and southern Chinese *C. a. auratus*. Values of *F*_ST_ among the goldfish from Hangzhou, Kunming, Changchun, Lanzhou, Beijing, Seoul, Toronto and Japan were low (0.0000–0.1805), and N_m_ values were always high (2.2698–infinity). The results of AMOVA also revealed no significant genetic divergence (Ф_CT_ = 0.236, *P*>0.5) among southern China (Hangzhou and Kunming), northern China (Changchun, Lanzhou and Beijing), Canada, South Korea and Japan.

### Genetic Diversity

Haplotype (H_d_) and nucleotide (π) diversity based on CR and *Cytb* sequences separately (Table 2) of sublineages C5 and C6 (CR: H_d_ = 0.9397±0.0040, π = 0.0929±0.0447; *Cytb*: H_d_ = 0.9541±0.0149, π = 0.0062±0.0033) were always higher than in goldfish (CR: H_d_ = 0.3782±0.0401, π = 0.0023±0.0018; *Cytb*: H_d_ = 0.1971±0.0384, π = 0.0007±0.0006). These results revealed a reduction of genetic diversity from native goldfish to aquaculture goldfish.

We also classified the goldfish by the three-breed taxonomy and subsequently compared their levels of genetic diversity. Haplotype h19 was shared by the Egg-goldfish and Wen-goldfish. Haplotypes Jap1 and Jap2 only occurred in Egg-goldfish, which have double tail fins and no dorsal fins. Six *Cytb* haplotypes were unique to Grass-goldfish and only haplotype B13 occurred in all three breeds. Haplotype B9 was detected in Wen-goldfish only. Grass-goldfish had a higher level of genetic diversity (Table 2) for *Cytb* (CR: H_d_ = 0.4436±0.0635, π = 0.0045±0.0029; *Cytb*: H_d_ = 0.4509±0.0684, π = 0.0017±0.0011) than either Egg-goldfish (CR: H_d_ = 0.6471±0.0458, π <0.0001; *Cytb*: all the specimens of Egg-goldfish shared the haplotype B13) or Wen-goldfish (CR: H_d_ = 0.1949±0.0667, π <0.0001; *Cytb*: H_d_ = 0.0230±0.0022, π <0.0001). These results revealed the loss of genetic diversity also occurred during the anthropogenic transition from Grass-goldfish to other breeds.

### Neutrality Tests

Values for Tajima’s *D* (CR: −1.083; *Cytb*: −2.266) and Fu and Li’s D* (CR: −0.871; *Cytb*: −6.423) and F* (CR: −1.139; *Cytb*: −5.703) were all negative in the goldfish and statistically significant (*P*<0.01) in our analysis based on the *Cytb* dataset (Table 2). Tajima’s *D* (CR: −0.162; *Cytb*: −1.943) and Fu and Li’s D* (CR: −0.636; *Cytb*: −4.737) and F* (CR: −0.560; *Cytb*: −4.423) values were also significantly negative in Grass-goldfish for the *Cytb* dataset (Table 2).

### Hypothesis-testing for Grouping Breeds

Four morphological constraint trees and the unconstrained best tree were represented in Figure S1. Results of the Templeton and SH tests were summarized in Table 4. The Templeton test rejected body-shape and eye-appearance (*P*<0.05) as being correlated with matrilineal history. P-values for the SH test showed that the best unconstrained ML topology differed significantly from the morphological constraint tree for eye-appearance (*P*<0.05). Differences in ln L values also revealed that the condition of the goldfish’s eye (normal or derived) was the least informative character for grouping by history (23.66), followed by body-shape (egg-shaped or slender, 14.22), and dorsal fin (retained or loss, 14.22). The number of tailfins (single or double, 4.61) was most indicative of genealogical history. Therefore, the three-breed scheme (Grass-goldfish, Wen-goldfish, and Egg-goldfish) better reflected history than either the four-breed or the five-breed systems that emphasized eye condition.

**Table 4:** Results of the Templeton and Shimodaira-Hasegawa tests for trees that constrain the primary morphological characteristics for different taxonomies of goldfish based on combined CR and *Cytb* datasets.

	Best tree L = 421	Difference in tree L	Templeton test (p)	Best tree –ln L = 3632.62	Difference in ln L	SH test (p)
Body shape(slender vs. egg-shaped)	431	10	0.0117*	3646.84	14.22	0.074
Dorsal-fin condition(retained vs. loss)	424	3	0.2668	3646.84	14.22	0.074
Eye-appearance(normal vs. derived)	429	8	0.0386*	3656.28	23.66	0.047*
Tailfin number(single vs. double)	428	7	0.0654	3637.23	4.61	0.154

* *P*<0.05.

Fisher’s exact test based on the concatenated *Cytb* and CR datasets obtained a highly significant (*P*<0.001) relationship between genetic variation sites and lineage. This indicated that the domestication of goldfish was constrained to particular matrilines.

## Discussion

### Origin of Goldfish

Our analyses suggest that Chinese goldfish have a matrilineal origin from native southern Chinese *C. a. auratus*, especially lineages from the lower Yangtze River. The genealogical analyses resolve the origin of goldfish from native Chinese *Carassius*, a finding consistent with that of Komiyama *et al.* [13]. The matrilineal genealogy (Figure 1) and *F*_ST_ values (Table 3) further indicate a much closer relationship between the goldfish and sublineages C5 and C6 of *C. a. auratus* from southern China rather than the gibel carp (sublineage C2) from northern China. This discovery differs from the suggestion of the origin of the goldfish being from the gibel carp [13]. Values of N_m_ (Table 3) also suggest strong gene flow occurs between goldfish and native *Carassius* from the lower Yangtze River. All analyses are consistent with the historical record, which suggests that Hangzhou and Jiaxin, Zhejiang, China might be the area of domestication [1]. Our analyses do not detect significant genetic divergence among the different regions where goldfish live; strong gene flow appears to occur among these regions. These results are not surprising considering the long history of commercialization, artificial selection, and hybridization among different breeds and regions of goldfish [1], [2], [4].

Other evidence excludes the gibel carp from being the ancestor of goldfish. Gibel carp are usually hexaploids with more than 150 chromosomes [45]. In contrast, goldfish are always tetraploids and have around 100 chromosomes [4], [46]. Further, the historical distribution of the gibel carp (*C. a. gibelio*) appears to be restricted to the northern Amur River system and Europe [47]–[50]. Historically, the distributions for goldfish and the gibel carp did not overlap. Therefore, our resolution of a southern origin for goldfish is valid not only because of the strength of the historical geography and ploidy levels of these fishes, but also because of the incontrovertible exclusion of the matrilines of the gibel carp.

### Domestication History of Goldfish

Anthropogenic selection of native *Carassius* eliminated aesthetically unappealing goldfish and this action appears to be responsible for the stepwise decrease in genetic diversity of domesticated goldfish, i.e. the loss of genetic variation from native goldfish to Grass-goldfish in aquiculture followed by further loss within remaining breeds of goldfish. A strong reduction of genetic diversity should accompany the domestication and this is seen as a recent bottleneck event or founder effects, which occurs in domesticated pigs [51], [52], maize [53], and rice [54]. Both the extraordinarily lower genetic diversity and the significantly negative results for the tests of Tajima’s *D* and Fu and Li’s D* and F* (Table 2) indicate founder effects and bottlenecking during the domestication of goldfish. Based on recorded history, native red *Carassius* were initially herding in ‘free life ponds’ at many temples in Hangzhou and Jiaxin, Zhejiang, China, and without anthropogenic breeding selection [1], [4]. The morphology of the Grass-goldfish is less derived and more similar to the native *Carassius* than other breeds [1], [7], [8]. Our analyses reveal that the Grass-goldfish has higher level of genetic diversity than either Egg-goldfish or Wen-goldfish. The *F*_ST_ values also indicate that Grass-goldfish and southern Chinese *C. a. auratus* differ less from each other than do either Egg-goldfish or Wen-goldfish from southern Chinese *C. a. auratus*. These findings indicate that the Grass-goldfish is likely the first domesticated breed of Chinese goldfish.

Strong anthropogenic selection for aesthetics is likely responsible for the further loss of genetic diversity among different breeds of goldfish. Our analyses for the three-breed system detect a further decrease in genetic diversity from Grass-goldfish to Egg- or Wen-goldfish (Table 2). Tajima’s *D* and Fu and Li’s D* and F* in Grass-goldfish are also significantly negative (Table 2). Wen-goldfish and Egg-goldfish both have many derived morphological features relative to the Grass-goldfish, such as the egg-shaped body, the possession of double tailfins, the absence of dorsal fins, and/or dragon-eyes [4], [6]–[8]. These findings correspond to anthropogenic selection to eliminate aesthetically unappealing goldfish and the consequential dramatic reduction in genetic diversity, which occurs in Wen- and Egg-goldfish in the three-breed system.

### Three-breed Taxonomy and the History of Domestication

Given the absence of a recorded history of the domestication of goldfish, we employed phylogenetic methods to reconstruct the past. Our analyses reveal that the three-breed taxonomy–Grass-goldfish, Egg-goldfish, and Wen-goldfish–better indicates the history of domestication than either the four-breed or the five-breed systems that emphasize eye-condition. The results of hypothesis-tests indicate that the condition of the fins is informative for grouping goldfish with respect to their evolutionary history. In the three-breed taxonomy, the Grass-goldfish and native *Carassius* differ only in the color of their scales [4], [6]–[8], and the condition of dorsal fin (loss or retained) distinguishes the Egg-breed from the Wen-breed [1]. Biomechanically, the dorsal fin functions to maintain balance when swimming. Without the dorsal fin, most fishes cannot stay upright. Dorsal finlessness appears after the attainment of double tailfins, which compensate for the loss of dorsal fins [55]. Therefore, Egg-goldfish (no dorsal fin) likely have a more recent origin than goldfish with double tailfins. The further examination of other genes closely related to the morphological characteristics of goldfish can test this prediction.

## Supporting Information

Figure S1 **Four morphological constraint-trees and the best unconstrained matrilineal genealogy for goldfish.** Unique haplotypes identified for each morphological characteristic were constrained to being monophyletic based on the concatenated *Cytb* and CR data, and using *Carassius carassius* as the outgroup taxon. The best unconstrained tree was shown at the bottom of the figure. Photographs of the goldfish for each morphological characteristic were mapped to the genealogy.
(TIF)

## References

[1] Wang CY (1985) The origin of the goldfish. Bull Biol 12: 11–12.

[2] Smartt J (2001) Goldfish Varieties and Genetics: Handbook for Breeders. Oxford: Blackwell Science. 216 p.

[3] Berg LS (1949) Freshwater Fishes of the USSR and Adjacent Countries, Part II. Moscow and Leningrad: Academy of Sciences, USSR. 467–925.

[4] Wang CY (2000) Chinese Goldfish. Beijing: Jindun Press. 81 p.

[5] Brewster B, Fletcher N (2004) Keeping Goldfish. Surrey: Interpet Publishing. 96 p.

[6] Eschmeyer, William N (1998) Catalog of Fishes. Special Publication of the Center for Biodiversity Research and Information, no. 1, vol 1–3. San Francisco: California Academy of Sciences. 2905 p.

[7] Wang CY (1983) How the different breeds of the goldfish formed. Bull Biol 3: 29–31.

[8] Li P (1959) Phylogeny relationship of different breeds of Chinese goldfish. Chinese J Zool 3: 248–251.

[9] Fu YY (1981) Initial viewpoint about the taxonomy of Chinese goldfish. Freshwater Fisheries 6: 15–18.

[10] Chen Z (1959) The Domestication and Variation of Goldfish. Beijing: Science Press. 31 p.

[11] Liang QJ, Peng YX (1994) An analysis of muscle proteins of the wild crucian (*Carassius auratus*) and five representative varieties of goldfishes (*C. auratus* var.) by electrophoresis methods. Zool Res 15: 68–75.

[12] Zhu XL, Wang ZY, Han ZQ (2010) The phylogenetic relationship between goldfish and crucian carp *Carassius auratus* in different regions based on mtDNA D-loop region sequences. Journal of Dalian Fisheries University 25: 153–157.

[13] Komiyama T, Kobayashi H, Tateno Y, Inoko H, Gojobori T, et al (2009) An evolutionary origin and selection process of goldfish. Gene 430: 5–11. 19027055 10.1016/j.gene.2008.10.019

[14] Gao Y, Wang SY, Luo J, Murphy RW, Du R, et al (2012) Quaternary palaeoenvironmental oscillations drove the evolution of the Eurasian *Carassius* auratus complex (Cypriniformes, Cyprinidae). J Biogeogr 39: 2264–2278.

[15] Tzeng CS, Hui CF, Shen AC, Huang PC (1992) The complete nucleotide sequence of the *Crossostoma lacustre* mitochondrial genome: conservation and variations among vertebrates. Nucleic Acids Res 20: 4853–4858. 1408800 10.1093/nar/20.18.4853 PMC334242

[16] Chang YS, Huang FL, Lo TB (1994) The complete nucleotide sequence and gene organization of carp (*Cyprinus carpio*) mitochondrial genome. J Mol Evol 38: 138–155. 8169959 10.1007/BF00166161

[17] Meyer A (1993) Evolution mitochondrial DNA in fishes. In: Molecular Biology Frontiers, Biochemistry and Molecular Biology of Fishes Vol 2, Hochachka PW, Mommsen TP, editors. The Hague: Elsevier Science Publishers. 1–38.

[18] Broughton RJ, Milam JE, Roe BA (2001) The complete sequence of the zebrafish (*Danio rerio*) mitochondrial genome and evolutionary patterns in vertebrate mitochondrial DNA. Genome Res 11: 1958–1967. 11691861 10.1101/gr.156801 PMC311132

[19] Moritz C, Dowling TE, Brown WM (1987) Evolution of animal mitochondrial DNA: relevance for population biology and systematics. Annu Rev Ecol Evol Syst 18: 269–292.

[20] Johns GC, Avise JC (1998) A comparative summary of genetic distance in the vertebrates from the mitochondrial cytochrome b. Mol Biol Evol 15: 1481–1490. 12572611 10.1093/oxfordjournals.molbev.a025875

[21] Kumar S, Dudley J, Nei M, Tamura K (2008) MEGA: a biologist-centric software for evolutionary analysis of DNA and protein sequences. Brief Bioinform 9: 299–306. 18417537 10.1093/bib/bbn017 PMC2562624

[22] Tamura K, Dudley J, Nei M, Kumar S (2007) MEGA4: molecular evolutionary genetics analysis (MEGA) software version 4.0. Mol Biol Evol 24: 1596–1599. 17488738 10.1093/molbev/msm092

[23] Xia X, Xie Z (2001) DAMBE: software package for data analysis in molecular biology and evolution. J Hered 92: 371–373. 11535656 10.1093/jhered/92.4.371

[24] Swofford DL (2002) PAUP*. Phylogenetic Analysis Using Parsimony (*and Other Methods). Ver. 4. Sunderland, Massachusetts: Sinauer Associates.

[25] Huelsenbeck JP, Ronquist F (2001) MRBAYES: Bayesian inference of phylogenetic trees. Bioinformatics 17: 754–755. 11524383 10.1093/bioinformatics/17.8.754

[26] Goldman N (1993a) Simple diagnostic statistical tests of models of DNA substitution. J Mol Evol 37: 650–661. 8114118 10.1007/BF00182751

[27] Goldman N (1993b) Statistical tests of models of DNA substitution. J Mol Evol 36: 182–198. 7679448 10.1007/BF00166252

[28] Huelsenbeck JP, Crandall KA (1997) Phylogeny estimation and hypothesis testing using maximum likelihood. Annu Rev Ecol Syst 28: 437–466.

[29] Posada D, Crandall KA (1998) MODELTEST: testing the model of DNA substitution. Bioinformatics 14: 817–818. 9918953 10.1093/bioinformatics/14.9.817

[30] Akaike H (1974) A new look in at statistical model identification. IEEE Transact Automatic Control 19: 716–722.

[31] Rambaut A, Drummond AJ (2009) Tracer v1.9. Available: http://beast.bio.ed.ac.uk/Tracer.

[32] Felsenstein J (1985) Confidence limits on phylogenies: an approach using the bootstrap. Evolution 39: 783–791. 28561359 10.1111/j.1558-5646.1985.tb00420.x

[33] Stamatakis A, Hoover P, Rougemont J (2008) A rapid bootstrap algorithm for the RAxML web-servers. Syst Biol 75: 758–771. 10.1080/10635150802429642 18853362

[34] Nakamura M (1969) Cyprinid Fishes of Japan. Studies on the life history of cyprinid fishes of Japan. Tokyo: Research Institute for Natural Resources. 455 p.

[35] Meng QW, Su JX, Miao XZ (1995) Fish Taxonomy. Beijing: China Agriculture Press. 1158 p.

[36] Eschmeyer WN (1998) Catalog of Fishes. Special Publication No.1, Centre of Biodiversity Research and Information. San Francisco: California Academy of Sciences.

[37] Luo J, Zhang YP, Zhu CL, Xiao WH, Huang SY (1999) Genetic diversity in crucian carp (*Carassius auratus*). Biochem Genet 37: 267–279. 10626035 10.1023/a:1018751008848

[38] Wheeler A (1977) The origin and distribution of the freshwater fishes of the British Isles. J Biogeogr 4: 1–24.

[39] Excoffier L, Laval G, Schneider S (2005) Arlequin (version 3.0): an integrated software package for population genetics data analysis. Evol Bioinform Online 1: 47–50. PMC2658868 19325852

[40] Tajima F (1989) Statistical method for testing the neutral mutation hypothesis by DNA polymorphism. Genetics 123: 585–595. 2513255 10.1093/genetics/123.3.585 PMC1203831

[41] Fu YX, Li WH (1983) Statistical tests of neutrality of mutations. Genetics 133: 693–709. 10.1093/genetics/133.3.693 PMC1205353 8454210

[42] Rozas J, Sanchez-DelBarrio JC, Messeguer X, Rozas R (2003) DnaSP, DNA polymorphism analyses by the coalescent and other methods. Bioinformatics 19: 2496–2497. 14668244 10.1093/bioinformatics/btg359

[43] Templeton AR (1983) Phylogenetic inference from restriction endonuclease cleavage sites maps with particular reference to the evolution of humans and the apes. Evolution 37: 221–244. 28568373 10.1111/j.1558-5646.1983.tb05533.x

[44] Shimodaira H, Hasegawa M (1999) Multiple comparisons of log-likelihoods with applications to phylogenetic inference. Mol Biol Evol 16: 1114–1116.

[45] Zhu HP, Ma DM, Gui JF (2006) Triploid origin of the gibel carp as revealed by 5S rDNA localization and chromosome painting. Chromosome Res 14: 767–776. 17115331 10.1007/s10577-006-1083-0

[46] Wang CY, Li YL (1982) Studies on the Karyotype of Goldfish (*Carassius auratus*) I. A comparative study of the chromosomes in crucian and red dragon-eye goldfish. J of Gen Genom 9: 238–242.

[47] Cherfas NB (1981) Gynogenesis in fishes. In: Genetic Bases of Fish Selection (ed Kirichnikov VS). Berlin: Springer-Verlag. 255–273.

[48] Jiang YG, Yu HX, Chen BD, Liang SC (1983) Biological effect of heterologous sperm on gynogenetic offspring in *Carassius auratus gibelio* . Acta Hydrobiol Sinica 8: 1–13.

[49] Gui JF (1997) Retrospectus and prospects of studies on the mechanism of natural gynogenesis in silver crucian carp (*Carassius auratus gibelio*). Bull Natl Natur Sci Found China 1: 11–16.

[50] Gui JF (2007) Genetic Basis and Artificial Control of Sexuality and Reproduction in Fish. Beijing: Science Press. 247 p.

[51] Li J, Yang H, Li J-r, Li HP, Ning T, et al (2010) Artificial selection of the melanocortin receptor 1 gene in Chinese domestic pigs during domestication. Heredity 105: 274–281. 20179735 10.1038/hdy.2009.191

[52] Ji YQ, Wu DD, Wu GS, Wang GD, Zhang YP (2011) Multi-locus analysis reveals a different pattern of genetic diversity for mitochondrial and nuclear DNA between wild and domestic pigs in East Asia. PLoS One 6: e26416. 22065995 10.1371/journal.pone.0026416 PMC3204973

[53] Wang RL, Stec A, Hey J, Lukens L, Doebley J (1999) The limits of selection during maize domestication. Nature 398: 236–239. 10094045 10.1038/18435

[54] Olsen KM, Caicedo AL, Polato N, McClung A, McCouch S, et al (2006) Selection under domestication: evidence for a sweep in the rice waxy genomic region. Genetics 173: 975–983. 16547098 10.1534/genetics.106.056473 PMC1526538

[55] Drucker EG, Lauder GV (2001) Locomotor function of the dorsal fin in teleost fishes: experimental analysis of wake forces in sunfish. J Exp Biol 204: 2943–2958. 11551984 10.1242/jeb.204.17.2943

