# Supplementary figures and images for: Origin of Chinese Goldfish and Sequential Loss of Genetic Diversity Accompanies New Breeds
Source: PLoS One. 2013 Mar 19;8(3):e59571. doi: 10.1371/journal.pone.0059571 (PMC3602300; doi:10.1371/journal.pone.0059571)

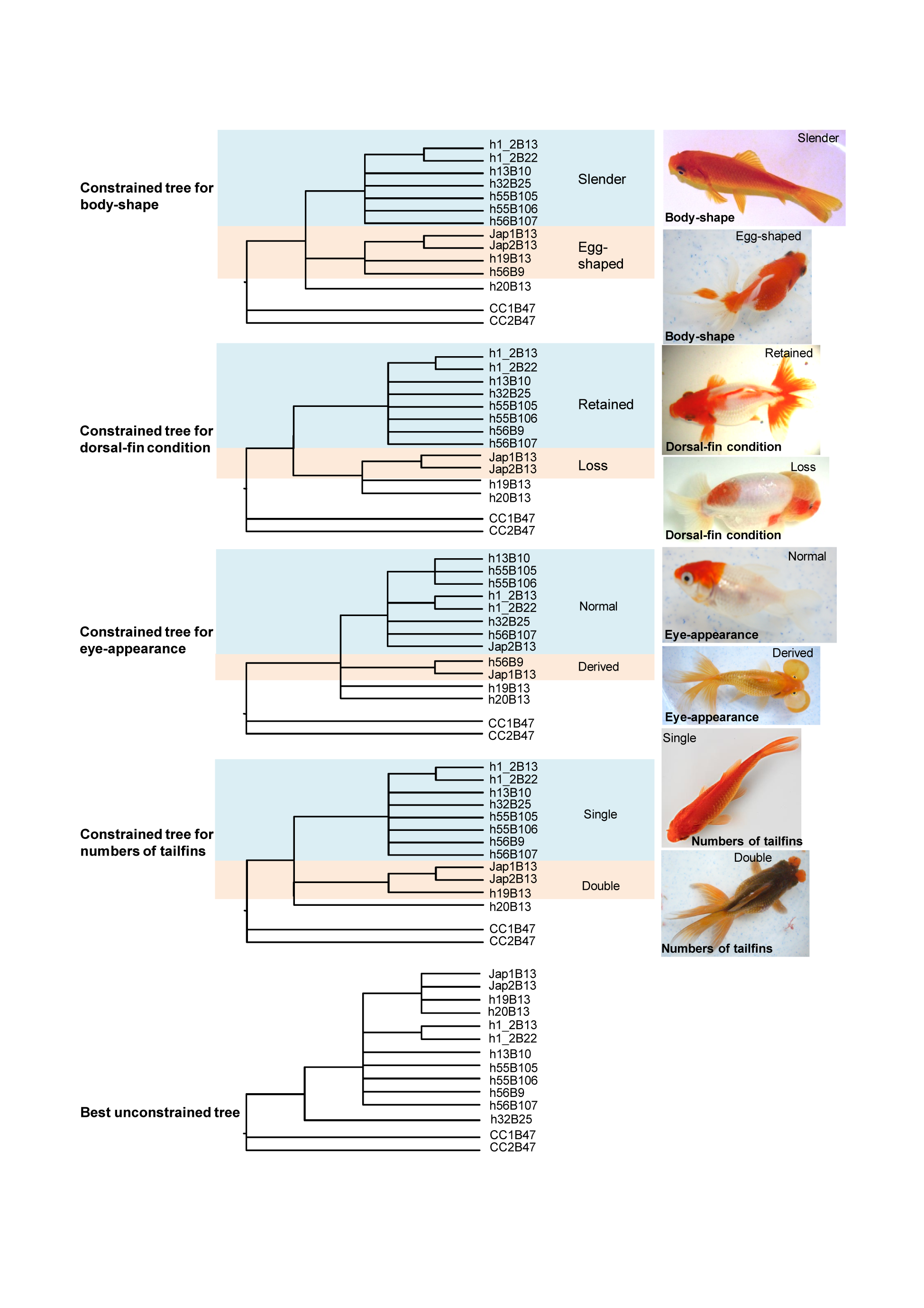

Supplement: Figure S1 — Four morphological constraint-trees and the best unconstrained matrilineal genealogy for goldfish. Unique haplotypes identified for each morphological characteristic were constrained to being monophyletic based on the concatenated Cytb and CR data, and using Carassius carassius as the outgroup taxon. The best unconstrained tree was shown at the bottom of the figure. Photographs of the goldfish for each morphological characteristic were mapped to the genealogy. (TIF) [file pone.0059571.s001.tif]
